# LONGITUDINAL ASSOCIATIONS OF HEALTH BEHAVIOR ADHERENCE WITH PSYCHOLOGICAL WELL-BEING AND NUMBER OF ILLNESSES

**DOI:** 10.1093/geroni/igae098.4234

**Published:** 2024-12-31

**Authors:** Fumiko Hamada, Soomi Lee

**Affiliations:** USF HPCC/USF School of Aging, Tampa, Florida, United States; The Pennsylvania State University, University Park, Pennsylvania, United States

## Abstract

Lifelong healthy habits are vital for healthy aging. This study examined the longitudinal associations of adherence to three key health behaviors (i.e., sleep, physical activity, healthy eating) on psychological well-being (PWB) and illnesses 10 years later. Data were from Midlife in the United States II and III (2005-2006, 2013-2017) participants who provided daily diary data at both timepoints (n=858; Mage=54 at T1). Over eight consecutive days, participants reported nightly sleep duration and the daily amount of vigorous physical activity. A subsample (n=428) reported weekly fast-food consumption. Adherence rates for each health behavior were calculated at each timepoint by summing the number of days with optimal sleep (6< sleep hours≤9), vigorous physical activity (≥30 minutes), and not eating fast food. Participants also completed a survey for PWB and the number of illnesses. A series of general linear or Poisson models examined the associations between the adherence variables and PWB and the number of illnesses, adjusting for sociodemographic and health covariates, baseline PWB or illnesses, and 10-year change in each adherence behavior. Adherence to optimal sleep and not eating fast food were stable across 10 years, while adherence to physical activity decreased. Greater adherence to optimal sleep (B=1.31; SE=0.45; p=0.003) and physical activity (B=1.16; SE=0.43; p=0.007) were each associated with better PWB 10 years later but not with the number of illnesses. Greater adherence to not eating fast food was associated with fewer illnesses (B=-0.12; SE=0.03; p< 0.001) but not PWB. Findings suggest that adherence to healthy lifestyles may be crucial for healthy aging.

